# Supplementary material for: Heat adaptation of phage T7 under an extended genetic code
Source: Virus Evol. 2021 Dec 1;7(2):veab100. doi: 10.1093/ve/veab100 (PMC8923235; doi:10.1093/ve/veab100)
Supplement: veab100_Supp [file veab100_supp.zip › Sequencing_Analysis.zip!/Sequencing_Analysis/A3/evidence/JC_68.html]

BRESEQ :: Evidence


| New junction evidence | | | | | | | | | | | |
| --- | --- | --- | --- | --- | --- | --- | --- | --- | --- | --- | --- |
|  | | seq id | position | reads (cov) | reads (cov) | score | skew | freq | annotation | gene | product |
| \* | ? | NC\_001604.1 | = 13354 | 265 (0.150) | 339 (0.200) | 173/594 | 4.9 | 53.9% | intergenic (–/–) | *–/–* | –/– |
|  | ? | NC\_001604.1 | 14356 = | 317 (0.180) |  |  |  |  | intergenic (–/–) | *–/–* | –/– |
| Rejected: Coverage evenness skew score above cutoff. | | | | | | | | | | | |

Only 100 of 348 total aligned reads displayed.  

`TGATACTATTATTGCCCTTGAGCGTAATCAGCAAGGCGATATGCCTAACCTTGTCCTCGTTCGTATTCTCAAGTGCCGCTTTACTGGTGATACTGGTATCGCTGGCTACATGGAATACAACAAGGAAACCGGATGGCTTGAACCATCAAGTTACTCAGGGGAAGAAGAGTCACACTCAGAGTCAACAGACTGGTCCAACGACACTGACTTCTGACAGGATTCTTGATGACTTTCCAGACGACTACGAGAAGTTTCGCTGGAGAGTCCCATTCTAATACGACTCACTAAAGGAGACA‑CACCATG‑‑‑‑‑‑‑‑‑‑‑‑‑‑‑‑‑‑‑‑‑‑‑‑‑‑‑‑‑‑‑‑‑‑‑‑‑‑‑‑‑‑‑‑‑‑‑‑‑‑‑‑‑‑‑‑‑‑‑‑‑‑‑‑‑‑‑‑‑‑‑‑‑‑‑‑‑‑‑‑‑‑‑‑‑‑‑‑‑‑‑‑‑‑‑‑‑‑‑‑‑‑‑‑‑‑‑‑‑‑‑‑‑‑‑‑‑‑‑‑‑‑‑‑‑‑‑‑‑‑‑‑‑‑‑‑‑‑‑‑‑‑‑‑‑‑‑‑‑‑‑‑‑‑‑‑‑‑‑‑‑‑‑‑‑‑‑‑‑‑‑‑‑‑‑‑‑‑‑‑‑‑‑‑‑‑‑‑‑‑‑‑‑‑‑‑‑‑‑‑‑‑‑‑‑‑‑‑‑‑‑‑‑‑‑‑‑‑‑‑‑‑‑‑‑‑‑‑‑‑‑‑‑‑‑‑‑‑‑‑‑‑‑‑‑‑‑‑‑‑‑‑‑‑‑‑‑‑‑‑‑‑‑‑‑‑‑‑‑‑‑‑‑‑‑‑‑‑‑‑‑‑‑‑‑‑‑‑‑‑‑‑‑‑‑‑‑‑  >  NC_001604.1/13052‑13354`  
`‑‑‑‑‑‑‑‑‑‑‑‑‑‑‑‑‑‑‑‑‑‑‑‑‑‑‑‑‑‑‑‑‑‑‑‑‑‑‑‑‑‑‑‑‑‑‑‑‑‑‑‑‑‑‑‑‑‑‑‑‑‑‑‑‑‑‑‑‑‑‑‑‑‑‑‑‑‑‑‑‑‑‑‑‑‑‑‑‑‑‑‑‑‑‑‑‑‑‑‑‑‑‑‑‑‑‑‑‑‑‑‑‑‑‑‑‑‑‑‑‑‑‑‑‑‑‑‑‑‑‑‑‑‑‑‑‑‑‑‑‑‑‑‑‑‑‑‑‑‑‑‑‑‑‑‑‑‑‑‑‑‑‑‑‑‑‑‑‑‑‑‑‑‑‑‑‑‑‑‑‑‑‑‑‑‑‑‑‑‑‑‑‑‑‑‑‑‑‑‑‑‑‑‑‑‑‑‑‑‑‑‑‑‑‑‑‑‑‑‑‑‑‑‑‑‑‑‑‑‑‑‑‑‑‑‑‑‑‑‑‑‑‑‑‑‑‑‑‑‑‑‑‑‑‑‑‑‑‑‑‑‑‑‑‑‑‑‑‑‑‑‑‑‑‑‑‑‑‑‑‑‑‑‑‑‑‑‑‑‑‑‑‑‑‑‑‑‑‑‑‑atgATCGTTTCTGACATCGAAGCTAACGCCCTCTTAGAGAGCGTCACTAAGTTCCACTGCGGGGTTATCTACGACTACTCCACCGCTGAGTACGTAAGCTACCGTCCGAGTGACTTCGGTGCGTATCTGGATGCGCTGGAAGCCGAGGTTGCACGAGGCGGTCTTATTGTGTTCCACAACGGTCACAAGTATGACGTTCCTGCATTGACCAAACTGGCAAAGTTGCAATTGAACCGAGAGTTCCACCTTCCTCGTGAGAACTGTATTGACACCCTTGTGTTGTCACGTTTGATTCATTCCA  >  NC_001604.1/14356‑14653`  

                                                                                                                                                                                                                                                                                                                                                                                                                                                                                                                                                                                                                            
`gagacagATTATTGCCCTTGAGCGTAATCAGCAAGGGGATATGCCTAACCTTGTCCTCGTTCGTATTCTCAAGTGCCGCTTTACTGGTGATACTGGTATCGCTGGCTACATGGAATACAACAAGGAAACCGGATGGCTTGAACCATCAAGTTACTCAGGGGAAGAAGAGTCACACTCAGAGTAAACAGACTGGTCCAACGACACTGACTTCTGACAGGATTCTTGATGACTTTCCAGACGACTACGAGAAGTTTCGCTGGAGAGTCCCATTCTAATACGACTCACTAAAGGAGACA‑CACCA                                                                                                                                                                                                                                                                                                              <  1:203319/294‑1`  
      `TATTATTGCCCTTGAGCGGAATCAGCAAGGCGATATGCCTAACCTTGTCCTCGTTCGTATTCTCAAGTGCCGCTTTACTGGTGATACTGGTATCGCTGGCTACATGGAATACAACAAGGAAACCGGATGGCTTGAACCATCAAGTTACTCAGGGGAAGAAGAGTCACACTCAGAGTCAACAGACTGGTCCAACGACACTGACTTCTGACAGGATTCTTGATGACTTTCCAGACGACTACGAGAAGTTTCGCTGGAGAGTCCCATTCTAATACGACTCACTAAAGGAGACA‑CACCATGATCG                                                                                                                                                                                                                                                                                                        <  1:10875/301‑1`  
      `TATTATTGCACTTGAGCGTAATCAGCAAGGCGATATGCCTAACCTTGTCCTCGTTCGTATTCTCAAGTGCCGCTTTACTGGTGATACTGGTATCGCTGGCTACATGGAATACAACAAGGAAACCGGATGGCTTGAACCATCAAGTTACTCAGGGGAAGAAGAGTCACACTCAGAGTCAACAGACTGGTCCAACGACACTGACTTCTGACAGGATTCTTGATGACTTTCCAGACGACTACGAGAAGTTTCGCTGGAGAGTCCCATTCTAATACGACTCACTAAAGGAGACA‑CACCATGATCG                                                                                                                                                                                                                                                                                                        <  1:168952/301‑1`  
       `ATTATTGCCCTTGAGCGTAATCAGCAAGGCGATATGCCTAACCTTGTCCTCGTTCGTATTCTCAAGTGCCGCTTTACTGGTGATACTGTTATCGCTGGCTACATGGAATACAACAAGGAAACCGAATGGCTTGAACCATCAAGTTACTCAGGGGAAGAAGAGTCACACTCAGAGTCAACAGACTGGTCCAACGACACTGACTTCTGACAGGATTCTTGATGACTTTCCAGACGACTACGAGAAGTTTCGCTGGAGAGTACCATTATATTACGACTCACTAAAGGAGAAA‑AACCATGATCGc                                                                                                                                                                                                                                                                                                       >  1:26803/1‑300`  
        `TTATTGCCCTTGAGCGTAATCAGCAAGGCGATATGCCTAACCTTGTCCTCGTTCGTATTCTCAAGTGCCGCTTTACTGGTGATACTGGTATCGCTGGCTACATGGAATACAACAAGGAAACCGGATGGCTTGAACCATCAAGTTACTCAGGGGAAGAAGAGTCACACTCAGAGTCAACAGACTGGTCCAACGACACTGACTTCTGACAGGATTCTTGATGACTTTCCAGACGACTACGAGAAGTTTCGCTGGAGAGTCCCATTCTAATACGCCTCACTAACGGAGACA‑CACCATGATCtgt                                                                                                                                                                                                                                                                                                      >  1:70247/1‑298`  
                  `TGAGCGTAATCAGCAAGGCGATATGCCTAACCTTGTCCTCGTTCGTATTCTCAAGTGCCGCTTTACTGGTGATACTGGTATCGCTGGCTACATGGAATACAACAAGGAAACCGGATGGCTTGAACCATCAAGTTACTCAGGGGAAGAAGAGTCACACTAAGAGTCAACAGACTGGTCCAACGACACTGACTTCTGACAGGATTCTTGATGACTTTCCAGACGACTACGAGAAGTTTCGCTGGAGAGTCCCATTCTAATACGACTCACTAAAGGAAACA‑CACCTTGATCGTTTCTGACATCG                                                                                                                                                                                                                                                                                            >  1:292761/1‑301`  
                   `GAGCGTAATCAGCAAGGCGATATGCCTAACCTTGTCCTCGTTCGTATTCTCAAGTGCCGCTTTACTGGTGATACTGGTATCGCTGGCTACATGGAATACAACAAGGAAACCGGATGGCTTGAACCATCAAGTTACTCAGGGGAAGAAGAGTCACACTCAGAGTCAACAGACTGGTCCAACGACACTGACTTCTGACAGGATTCTTGATGACTTTCCAGACGACTACGAGAAGTTTCGCTGGAGAGTCCCATTCTAATACGACTCACTAAAGGAGACA‑CACCATGATCGTTTCTGACATCGA                                                                                                                                                                                                                                                                                           >  1:40696/1‑301`  
                    `AGCGTAATCAGCAAGGCGATATGCCTAACCTTGTCCTCGTTCGTATTCTCAAGTGCCGCTTTACTGGTGATACTGGTATCGCTGGCTACATGGAATACAACAAGGAAACCGGATGGCTTGAACCATCAAGTTACTCAGGGGAAGAAGAGTCACACTCAGAGTCAACAGACTGGTACAACGACACTGACTTCTGACAGGATTCTTGATGACTTTCCAGACGACTACGAGAAGTTTCGCTGGAGAGTCCCATTCTAATACGACTCACTAAAGGAACCA‑CACCATGATCGTTTCTGACATCCAA                                                                                                                                                                                                                                                                                          >  1:231859/1‑301`  
                      `CGTAATCAGCAAGGCGATATGCCTAACCTTGTCCTCGTTCGTATTCTCAAGTGCCGCTTTACTGGTGATACTGGTATCGCTGGCTACATGGAATACAACAAGGAAACCGGATGGCTTGAACCATCAAGTTACTCAGGGGAAGAAGAGTCACACTCAGAGTCAACAGACTGGTCCAACGACACTGACTTATGACAGGATTCTTGATGACTTTCCAGACGACTACGAGAAGTTTCGCTGGCGAGTCCCATTCTAATACGACTCACTAAAGGAGACA‑CACCATGATCGTTTCTGACATCGAAGC                                                                                                                                                                                                                                                                                        <  1:157000/301‑1`  
                       `GTAATCAGCAATGCGATATGCCTAACCTTGTCCTCGTTCGTATTCTCAAGTGCCGCTTTACTGGTGATACTGGTATCGCTGGCTACATGGAATACAACAAGGAAACCGGATGGCTTGAACCATCAAGTTACTCAGGGGAAGAAGAGTCACACTCAGAGTCAACAGACTGGTCCAACGACACTGACTTCTGACAGGATTCTTGATGACTTTCCAGACGACTACGAGAAGTTTCGCTGGAGAGTCCCATTCTAATACGACTCACTAAAGGAGACA‑CACCATGATCGTTTCTGACATCGAAGCT                                                                                                                                                                                                                                                                                       >  1:177206/1‑301`  
                            `aaataggggGATATGCCCAACCTTTTCCTCGTTCGTATTCGTAAGTGCCGCTTTAATGGTGATACTGGTATCGCTGGCTACAAGGAATACAACAAGGAAACCGGATGGCTTGAACCATCAAGTTACTCAGGGGAAGAAGAGTCACACTCAGAGTCAACAGACTGGTCCAACGACACTGACTTCTGACAGGATTCTTGATGACTTTCCAGACGACTACGAGAAGTTTCGCTGGAGAGTCCCATTCTAATACGACTCACTAAAGGAGACA‑CACCATGATCGTTTCTGACATCGAAGCTAACGC                                                                                                                                                                                                                                                                                  <  1:206879/292‑1`  
                            `CAGCAAGGCGATATGCCTAACCTTGTCCTCGTTCGTATTCTCAAGTGCCGCTTTACTGGTGATACTGGTATCGCTGGCTACATGGAATACAACAAGGAAACCGGATGGCTTGAACCATCAAGTTACTCAGGGGAAGAAGAGTCACACTCAGAGTCAACAGACTGGTCCAACGACACTGACTTCTGACAGGATTCTTGATGACTTTCCAGACGACTACGAGAAGTTTCGCTGGAGAGTCCCATTCTAATACGACTCACTAAAGGAGACA‑CACCATGATCGctgtctcttatacacatctccg                                                                                                                                                                                                                                                                                  >  1:257526/1‑279`  
                              `GCAAGGTGATATGCCTAACCTTGTCCTCGTTCGTATTCTCAAGTGCCGCTTTACTGGTGATACTGGTATCGCTGGCTACATGGAATACAACAAGGAAACCGGATGGCTTGAACCATCAAGTTACTCAGGGGAAGAAGAGTCACACTCAGAGTCAACAGACTGGTCCAACGACACTGACTTCTGACAGGATTCTTGATGACTTTCCAGACGACTACGAGAAGTTTCGCTGGAGAGTCCCATTCTAATACGACTCACTAAAGGAGACA‑CACCATGATCGTTTCTGACATCGAAGCTAACGCCC                                                                                                                                                                                                                                                                                >  1:269928/1‑301`  
                                  `GGCGATATGCCTAACCTTGTCCTCGTTCGTATTCTCAAGTGCCGCTTTACTGGTGATACTGGTATCGCTGGCTACATGGAATACAACAAGGAAACCGGTTGGCTTGAACCATCAAGTTACTCAGGGGAAGAAGAGTCACACTCAGAGTCAACAGATTGGTCCAACGACACTGACTTCTGACAGGATTCTTGATGACTTTCCAGACGACTACGAGAAGTTTCGCTGGAGAGTCCCATTCTAATACGACTCACTAAAGGAGACA‑CACCATGATCGTTTCTGACATCGAAGCTAACACCCTCTT                                                                                                                                                                                                                                                                            >  1:117870/1‑301`  
                                      `ATATGCCTAACCTTGTCCTCGTTCGTATTCTCAAGTGCCGCTTTACTGGTGATACTGGTATCGCTGGCTACATGGAATACAACAAGGAAACCGGATGGCTTGAACCATCAAGTTACTCAGGGGAAGAAGAGTCACACTCAGAGTCAACAGACTGGTCCAACGACACTGACTTCTGACAGGATTCTTGATGACTTTCCAGACGACTACGAGAAGTTTCGCTGGAGAGTCCCATTCTAATACGACTCACTAAAGGAGACA‑CACCATGATCGTTTCTGACATCGAAGCTAACGCCCTCTTAGAG                                                                                                                                                                                                                                                                        <  1:184157/301‑1`  
                                       `aggGCCTAACCTTGTCCTCGTTCGTATTCTCAAGTGCCGCTTTACTGGTGATACTGGTATCGCTGGCTACATGGAATACAACAAGGAAACCGGATGGCTTGAACCATCAAGTTACTCAGGGGAAGAAGAGTCACACTCAGAGTCAACAGACTGGTCCAACGACACTGACTTCTGACAGGATTCTTGATGACTTTCCAGACGACTACGAGAAGTTTCGCTGGAGAGTCCCATTCTAATACGACTCACTAAAGGAGACA‑CACCATGATCGTTTCTGACATCGAAGCTAACGCCCTCTTAGAGA                                                                                                                                                                                                                                                                       <  1:103780/298‑1`  
                                        `attCCTATCCTTGTCCTCGTTCGTATTCTCAAGTGCCGCTTTACTGGTGATACTGGTATCGCTGGCTACATGGAATACAACAAGGAAACCGGATGGCTTGAACCATCAAGTTACTCAGGGGAAGAAGAGTCACACTCAGAGTCAACAGACTGGTCCAACGACACTGACTTCTGACAGGATTCTTGATGACTTTCCAGACGACTACGAGAAGTTTCGCTGGAGAGTCCCATTCTAATACGACTCACTAAAGGAGACA‑CACCATGATCGTTTCTGACATCGAAGCTAACGCCCTCTTAGAGAG                                                                                                                                                                                                                                                                      <  1:272277/298‑1`  
                                        `ATGCCTAACCTTGTCCTCGTTCGTATTCTCAAGTTCCGCTTTACTGGTGATACTGGTATCGCTGGCTACATGGAATACAACAAGGAAACCGGATGGCTTGAACCATCAAGTTACTCAGGGGAAGAAGAGTCACACTCAGAGTCAACAGACTGGTCCAACGACACTGACTTCTGACAGGATTCTTGATGACTTTCCAGACGACTACGAGAAGTTTCGCTGGAGAGTCCCATTCTAATACGACTCACTAAAGGAGACA‑CACCATGATCGTTTCTGACATCGAAGCTAACGCCCTCTTAGAGAG                                                                                                                                                                                                                                                                      >  1:57368/1‑301`  
                                        `ATGCCTAACCTTGTCCTCGTTCGTATTCGAAAGTGCCGCTTTACTGGTGATACTGGTATCACTGGCTACATGGAATACAACAAGGAAACCGGATGGCTTGAACCATCAAGTTACTCAGGGGAAGAAGAGTCACACGCAGAGTCAACAGACTGGTCCAACGACACTGACTTCTGATAGGATTCTTGATGACTTTCCAGACGACTACGAGAAGTTTCGCTGGAGAGTCCCATTCTAATACGACTCACTAAAGGAGACA‑CACCATGATCGTTTCTGACATCGAAGCCAACGCCATCTTAGAGAG                                                                                                                                                                                                                                                                      <  1:239084/301‑1`  
                                               `ACCTTGTCCTCGTTCGTATTCTCAAGTGCCGCTTTACTGGTGATACTGGTATCGCTGGCTACATGGAATACAACAAGGAAACCGGATGGCTTGAACCATCAAGTTACTCAGGGGAAGAAGAGTCACACTCAGAGTCAACAGACTGGTCCAACGACACTGACTTCTGACAGGATTCTTGATGACTTTCCAGACGACTACGAGAAGTTTCGCTGGAGAGTCCCATTCTAATACGACTCACTAAAGGAGACA‑CACCATGATCGTTTCTGACATCGAAGCTAACGCCCTCTTAGAGctgtctctt                                                                                                                                                                                                                                                               >  1:261411/1‑292`  
                                               `ACCTTGTCCTCGTTCGTATTCTCAAGTGCCGCTTTACTGGTGATACTGGTATAGCTGGCTACATGGAATACACCAAGGTAACCGGATGGCTTGAACCATCTAGTTACTCAGGGGAAGAAGAGTCACACTCATAGTCAGCAGACTGGTCCAACGACACTGCCTTCTGACAGGATTCTTGATGACTTTCCAGACGACTACGAGCTGTTTCGATGGCGAGTCACATTCTCTTATGACTCACTAAAGGAGACA‑CACCATGATCGTTTCTGACCTCGAAGCTACTGCCATCTTAGAGAGAGTaatt                                                                                                                                                                                                                                                               >  1:80889/1‑297`  
                                                `aCTTGTCCTCGTTCGTATTATCAAGTGCCGCTTTACTGGTGATACTGGTAACGCTGGCTACATGGAATACAACGAGGAAACCGGATGGCTTGAACCATCAAGTTACTCAGTGGAAGAAGAGTCACACTCAGAGTCAACAGACTGGTCCAACGACACTGACTTCTGACAGGATTCTTGATGACTTTCCAGACGACTACGAGAAGTTTCGCTGGAGAGTCCCATTCTAATACGACTCACTAAAGGAGACA‑CACCATGATCGTTTCTGACATCGAAGCTAACGCCCTCTTAGAGAGCGTCACTA                                                                                                                                                                                                                                                              <  1:213242/300‑1`  
                                                   `TGTCCTCGTTCGTATTCTCAAGTGCCGCTTTACTGGTGATACTGGTATCGCTGGCTACATGGAATACAACAAGGAAACCGGATGGCTTGAACCATCAAGTTACTCAGGGGAAGAAGAGTCACACTCAGAGTCAACAGACTGGTCCAACGACACTGACTTCTGACAGGATTCTTGATGACTTTCCAGACGACTACGAGAAGTTTCGCTGGAGAGTCCCATTCTAATACGACTCACTAAAGGAGACA‑CACCATGATCGTTTCTGACATCGAAGCTAACGCCCTCTTAGAGAGCGTCACTAAGT                                                                                                                                                                                                                                                           <  1:122772/301‑1`  
                                                    `GTCCTCGTTCGTATTCTCAAGTGCCGCTTTACTGGTGATACTGGTATCGCTGGCTACATGGAATACAACAAGGAAACCGGATTGCTTGAACCATCAAGTTACTCAGGGGAAGAAGAGTCACACTCAGAGTCAACAGACTGGTCCAACGACACTGACTTCTGACATGAATCTTGATGACTTTCCAGACGACTACGAGAAGTTTCGCTGGAGAGTCCCATTCTAATACGACTCACTAAAGGAGACA‑CACCATGATCGTTTCTGACATCGAAGCTAACGCCCTCTTAGAGAGCGTCACTActgt                                                                                                                                                                                                                                                          >  1:5388/1‑297`  
                                                    `GTCCTCGTTCGTATTCTCAAGTGCCGCTTTACTGGTGATACTGGTATCGCTGGCTACATGGAATACAACAAGGAAACCGGATGGCTTGAACCATCAAGTTACCCAGGGGAAGAAGAGTCACACTCAGAGTCAACAGACTGGTCCAACGACACTGACTTCTGACAGGATTCTTGATGACTTTCCAGACGACTACGAGAAGTTTCGCTGGAGAGTCCCATTCTACTACGACTCACTAAAGGAGACA‑CACCATGATCGTTTCTGACATCGAAGCTAACGTCCTCTTAGAGAGCGTCACTAAGTT                                                                                                                                                                                                                                                          >  1:115633/1‑301`  
                                                          `GTTCGTATTCTCAAGTGCCGCTTTACTGGTGATACTGGTATCGCTGGCTACATGGAATACAACAAGGAAACCGGATGGCTTGAACCATCAAGTTACTCATGGGAAGAAGAGTCACACTCAGAGTCAACAGACTGGTCCAACGACACTGACTTCTGACAGGATTCTTGATGACTTTCCAGACGACTACGAGAAGTTTCGCTGGAGAGTCCCATTCTAATACGACTCACTAAAGGATACA‑CACCATGATCGTTTCTGACATCGAAGCTAACGCCCTCTTAGAGAGCTTCACTAAGTTCAACTG                                                                                                                                                                                                                                                    <  1:2976/301‑1`  
                                                               `TATTCTCAAGTGCCGCTTTACGGGTGATACTGGTATCGCTGGCTACAAGGAATACAACAAGGAAACCGGATGGCTTGAACCATCAAGTTACTCAGGGGAAGAAGAGTCACACTCAGAGTCAACAGACTGGTCCAACGACACTGACTTCTGACAGGATTCTTGATGACTTTCCAGACGACTACGAGAAGTTTCGCTGGAGAGTCCCATTCTAATACGACTCACTAAAGGAGACA‑CACCATGATCGTTTCTGACATCGAAGCTAACGCCCTCTTAGAGAGCGTCACTAGGTTCCACTGCGGGG                                                                                                                                                                                                                                               <  1:163093/301‑1`  
                                                                  `accCAAGTGCCGCATTACTGGTGATACTGGTATCACTTGCTACATAGAATAAAAAAAGGAATCCGGATGGCTTGAACAATCAAGTTACTCAGGGGAAGAATAGTCACAATCAGAGTCAACAGACTGGTCCAACGACACTGAACTCTGACAGGATTCTTGATGACTTTCCAGACGACTGCGAGAAGTTTCGCTGGAGAGTCCCATTCTAATAAGACTCACTAAAGGAGACA‑CACCATGATCGTTTCTGACATCGAAGCTAACGCCCTCTTAGAGAGCGTCACTAAGTTCCACTGCAGGGTTA                                                                                                                                                                                                                                            <  1:93548/298‑1`  
                                                                       `AGTGCCGCTTTACTGGTGATACTGGTATCGCTGGCTACATGGAATACAACAAGGAAACCGGATGGCTTGAACCATCAAGTTACTCAGGGGAAGAAGAGTCACACTAAGAGTCAACAGACTGGTCCAACGACACTGACTTCTGACAGGATTCTTGATGACTTTCCAGACGACTACGAGAAGTTTCGCTGGAGAGTCCCATTCTAATACGACTCACTAAAGGAGACA‑CACCATGATCGTTTCTGACATCGAAGCTAACGCCCTCTTAGAGAGCGTCACTAAGTTCCACTGCGGGGTTATCTAC                                                                                                                                                                                                                                       <  1:222633/301‑1`  
                                                                                `TTACTGGTGATACTGGTATCGCTGGCTACATGGAATACAACAAGGAAACCGGATGGCTTGAACCATCAAGTTACTCAGGGGAAGAAGAGTCACACTCAGAGTCAACAGACTGGTCCAACGACACTGACTTCTGACAGGATTCTTGATGACTTTCCAGACGACTACGAGAAGTTTCGCTGGAGAGTCCCATTCTAATACGACTCACTAAAGGAGACA‑CACCATGATCGTTTCTGCCATCGAAGCTAACGCCCTCTTAGAGAGCGTCACTAAGTTCCACTGCGGGGTTATCTACGACTACTCC                                                                                                                                                                                                                              >  1:328776/1‑301`  
                                                                                       `gGATACTGGTATCGCTGGCTACATGGAATACAACAAGGAAACCGGATGGCTTGAACCATCAAGTTACTCAGGGGAAGAAGAGTCACACTCAGAGTCAACAGACTGGTCCAACGACACTGACTTCTGACAGGATTCTTGATGACTTTCCAGACGACTACGAGAAGTTTCGCTGGAGAGTCCCATTCTAATACGACTCACTAAAGGAGACA‑CACCATGATCGTTTCTGACATCGAAGCTAACGCCCTCTTAGAGAGCGTCACTAAGTTCCACTGCGGGGTTATCTACGACTACTCCACCGCTG                                                                                                                                                                                                                       <  1:321404/300‑1`  
                                                                                             `gGGTATCGCTGGCTACATGGAATACAACAAGGAAACCGGATGGCTTTAACCATCAAGTTACTCAGGGGAAGAAGAGTCACACTCAGAGTCAACAGACTGGTCCAACGACACTGACTTCTGACAGGATTCTTGATGACTTTCCAGACGACTACGAGAAGTTTCGTTGGAGAGTCCCATTCTAATACTACTCACTAAAGGAGACA‑CACCATGATCGTTTCTGACATCGAAGCTAACGCCCTCTTAGAGAGCGTCACGAAGTTCCACTGCGGGGTTATCTACGACTACTCCACCGCTGAGTACG                                                                                                                                                                                                                 <  1:139132/300‑1`  
                                                                                               `GTATCGCTGGCTACATGGAATACAACAAGGAAACCGGATGGCTTGAACCATCAAGTTACTCAGGGGAAGAAGAGTCACACTCAGAGTCAACAGACTGGTCCAACGACACTGACTTCTGACAGGATTCTTGATGACTTTCCAGACGACTACGAGAAGTTTCGCTGGAGAGTCCCATTCTAATACGACTCACTAAAGGAGACA‑CACCATGATCGTTTCTGACATCGAAGCTAACGCCCTCTTAGAGAGCGTCACTAAGTTCCACTGCGGGGTTATCTACGACTACTCCACCGCTGAGTACGTA                                                                                                                                                                                                               <  1:224422/301‑1`  
                                                                                                `TATCGCTGGCTACATGGAATACAACAAGGAAACCGGATGGCTTGAACCATCAAGTTACTCAGGGGAAGAA‑AGTCACACTCAGAGTCAACAGACTGGTCCAACGACACTGACTTCTGACAGGATTCTTGATGACTTTCCAGACGACTACGAGAAGTTTCGCTGGAGAGTCCCATTCTAATACGACTCACTAAAGGAGACA‑CACCATGATCGTTTCTGACATCGAAGCTAACGCCCTCTTAGAGAGCGTCACTAAGTTCCACTGCGGGGTTATCTACGACTACTCAACCGCTGAGTACGTAAG                                                                                                                                                                                                             <  1:247625/301‑1`  
                                                                                                 `ATCGCTGGCTACATGGAATACAACAAGGAAACCGGATGGCTTGAACCATCAAGTTACTCAGGGGAAGAAGAGTCACACTCAGAGTCAACAGACTGGTCCAACGACACTGACTTCTGACAGGATTCTTGATGACTTTCCAGACGACTACGAGAAGTTTCGCTGGAGAGTCCCATTCTAATACGACTCACTAAAGGAGACA‑CACCATGATCGTTTCTGACATCGAAGCTAACGCCCTCTTAGAGAGCGTCACTAAGTTCCACTGCGGGGTTATCTACGACTACTCCACCGCTGAGTACGTAAG                                                                                                                                                                                                             <  1:83841/301‑1`  
                                                                                                   `cgaTGGCTACATGGAATACAACAAGGAAACCGGATGGCTTGAACCATCAAGTTACTCAGGGGAAGAAGAGTCACACTCAGAGTCAACAGACTGGTCCAACGACACTGACTTCTGACAGGATTCTTGATGACTTTCCAGACGACTACGAGAAGTTTCGCTGGAGAGTCCCATTCTAATACGACTCACTAAAGGAGACA‑CACAATGATCGTTTCTGACATCGAAGCTAACGCCCTCTTAGAGAGCGTCACGAAGTTCCACTGCGGGGTTATCTACGACTACTCCACCGCTGAGTACGTAAGCT                                                                                                                                                                                                           <  1:106106/298‑1`  
                                                                                                             `ATGGAATACAACAAGGAAACCGGATGGCTTGAACCATCAAGTTACTCAGGGGAAGAAGAGTCACACTCAGAGTCAACAGACTGGTCCAACGACACTGACTTCTGACAGGATTCTTGATGACTTTCCAGACGACTACGAGAAGTTTCGCTGGAGAGTCCCATTCTAATACGACTCACTAAAGGAGACA‑CACCATGATCGTTTCTGACATCGAAGCTAACGCCCTCTTAGAGAGCGTCACTAAGTTCCACTTCGGGTCTATCTACGACTACTCTACCGCTGAGTACGTAAGCTACCGTCCGAG                                                                                                                                                                                                 >  1:14107/1‑301`  
                                                                                                                `GAATACAACAAGGAAACCGGATGGCTTGAACCATCAAGTTACTCAGGGGAAGAAGAGTCACACTCAGAGTCAACAGACTGGTCCAACGACACTGACTTCTGACAGGATTCTTGATGACTTTCCAGACGACTACGAGAAGTTTCGCTGGAGAGTCCCATTCTAATACGACTCACTAAAGGAGACA‑CACCATGATCGTTTCTGACATCCAAGCTAACGCCCTCTTAGAGAGCGTCACTAAGTTCCACTGCGGGGTTATCTACGACTACTCCACAGCTGCGTACGTAAGCTACCGTCCacgtta                                                                                                                                                                                              >  1:276395/1‑295`  
                                                                                                                        `aAAGGAAACCGGATGGCTTGAACCATCAAGTTACTCAGGGGAAGAAGAGTCACACTCAGAGTCAACAGACTGGTCCAACGACACTGACTTCTGACAGGATTCTTGATGACTTTCCAGACGACTACGAGAAGTTTCGCTGGAGAGTCCCATTCTAATACGACTCACTAAAGGAGACA‑CACCATGATCGTTTCTGACATCGAAGCTAACGCCCTCTTAGAGAGCGTCACTAAGTTCCACTGCGGGGTTATCTACGACTACTCCACCGCTGAGTACGTAAGCTACCGTCCGAGTGACTTCGGTG                                                                                                                                                                                      <  1:50886/300‑1`  
                                                                                                                           `GGAAACCGGATGGCTTGAACCATCAAGTTACTCAGGGGAAGAAGAGTCACACTCAGAGTCAACAGACTGGTCCAACGACACTGACTTCTGACAGGATTCTTGATGACTTTCCAGACGACTACGAGAAGTTTCGCTGGAGAGTCCCATTCTAATACGACTCACTAAAGGAGACA‑CACCATGATCGTTTCTGACATCGAAGCTAACGCCCTCTTAGAGAGCGTCACTAAGTTCCACTGCGGGGTTATCTACGACTACTCCACCGCTGAGTACGTAAGCTACCGTCCGAGTGACTTCGGTTCGT                                                                                                                                                                                   >  1:30098/1‑301`  
                                                                                                                                    `ATGGCTTGAACCATCAAGTTACTCAGGGGAAGAAGAGTCACACTCAGAGTCAACAGACTGGTCCAACGACACTGACTTCTGACAGGATTCTTGATGACTTTCCAGACGACTACGAGAAGTTTCGCTGGAGAGTCCCATTCTAATACGACTCACTAAAGGAGACA‑CACCATGATCGTTTCTGACATCGAAGCTAACGCCCTCTTAGAGAGCGTCACTAAGTTCCACTGCGGGGTTATCTACGACTACTCCACAGCTGAGTACGTAAGCTACCGTCCGAGTGACTTCGGTGCGTATCTGGATG                                                                                                                                                                          <  1:196799/301‑1`  
                                                                                                                                        `CTTGAAACATCAAGTTACTCAGGGGAAGAAGAGTCACACTCAGAGTCAACAGACTGGTCCAACGACACTGACTTCTGACAGGATTCTTGATGACTTTCCAGACGACTACGAGAAGTTTCGCTGGAGAGTCCCATTCTAATACGACTCACTAAAGGAGACA‑CACCATGATCGTTTCTGACATCGAAGCTAACGCCCTCTTAGAGAGCGTCACTAAGTTCCACTGCGGGGTTATCTACGACTACTCCACCGCTGAGTACGTAAGCTACCGTCCGAGTGACTTCGGTGCGTATCTGGATGCGCT                                                                                                                                                                      <  1:38435/301‑1`  
                                                                                                                                           `GAACCATCAAGTTACTCAGGGGAAGAAGAGTCACACTCAGAGTCAACAGACTGGTCCAACGACACTGACTTCTGACAGGATTCTTGATGACTTTCCAGACGACTACGAGAAGTTTCGCTGGAGAGTCCCATTCTAATACGACTCACTAAAGGAGACA‑CACCATGATCGTTTCTGACATCGAAGCTAACGCCCTCTTAGAGAGCGTCACTAAGTTCCACTGCGGGGTTATCTACGACTACTCCACCGCTGAGTACGTAAGCTACCGTCCGAGTGctgtctcttatacacatctccgagccca                                                                                                                                                                   >  1:219208/1‑273`  
                                                                                                                                            `atgtgtataagagacagGGGGAAGAAGAGTCACACTCAGAGTCAACAGACTGGTCCAACGACACTGACTTCTGACAGGATTCTTGATGACTTTCCAGACGACTACGAGAAGTTTCGCTGGAGAGTCCCATTCTAATACGACTCACTAAAGGAGACA‑CACCATGATCGTTTCTGACATCGAAGCTAACGCCCTCTTAGAGAGCGTCACTAAGTTCCACTGCGGGGTTATCTACGACTACTCCACCGCTGAGTACGTAAGCTACCGTCCGAGTGACTTCGGTGCGTATCTGGATGCGCTGGAA                                                                                                                                                                  <  1:87534/284‑1`  
                                                                                                                                                    `cGTTCCTCAGGGGAAGAAGAGTCACACTCAGAGTCAACAGACTGGTCCAACGACACTGACTTCTGACAGGATTCTTGATGACTTTCCAGACGACTACGAGAAGTTTCGCTGGAGAGTCCCATTCTAATACGACTCACTAAAGGAGACA‑CACCATGATCGTTTCTGACATCGAAGCTAACGCCCTCTTAGAGAGCGTCACTAAGTTCCACTGCGGGGTTATCTACGACTACTCCACCGCTGAGTACGTAAGCTACCGTCCGAGTGACTTCGGTGCGTATCTGGATGCGCTGGAAGCCGAGGT                                                                                                                                                          <  1:109559/300‑1`  
                                                                                                                                                         `agatgtgtataagagacagCACTCAGAGTCAACAGACTGGTCCAACGACACTGACTTCTGACAGGATTCTTGATGACTTTCCAGACGACTACGAGAAGTTTCGCTGGAGAGTCCCATTCTAATACGACTCACTAAAGGAGACA‑CACCATGATCGTTTCTGACATCGAAGCTAACGCCCTCTTAGAGAGCGTCACTAAGTTCCACTGCGGGGTTATCTACGACTACTCCACCGCTGAGTACGTAAGCTACCGTCCGAGTGACTTCGGTGCGTATCTGGATGCGCTGGAAGCCGAGGTTGCAC                                                                                                                                                     <  1:318122/282‑1`  
                                                                                                                                                              `GGGAAGAAGAGTCACACTCAGAGTCAACAGACTGGTCCAACGACACTGACTTCTGACAGGATTCTTGATGACTTTCCAGACGACTACGAGAAGTTTCGCTGGAGAGTCCCATTCTAATACGACTCACTAAAGGAGACA‑CACCATGATCGTTTCTGACATCGAAGCTAACGCCCTCTTAGAGAGCGTCACTAAGTTCCACTGCGGGGTTATCTACGACTACTCCACCGCTGAGTACGTAAGCTACCGTCCGAGTTACTTCGGTGCGTATCTGGATGCGCTGGAAGCCGAGGTTGCACGAGtc                                                                                                                                                >  1:17273/1‑299`  
                                                                                                                                                                  `AGAAGAGTCACACTCAGAGTCAACAGACGGGTCCAACGACACTGACTTCGGACAGGATTCTTGATGACTTTCCAGACGACTACGAGAAGTTTCGCTGGAGAGTCCCATTCTAATACGACTCACTAAAGGAGACA‑CACCATGATCGTTTCTGACATCGAAGCTAACGCCCTCTTAGAGAGCGTCACTAAGTTCCACTGCGGGGTTATCTACGACTACTCCACCGCTGAGTACGTAAGCTACCGTCCGAGTGACTTCGGTGCGTATCTGGATGCGCTGGAAGCCGAGGTTGCACGAGGCGGTC                                                                                                                                            <  1:77999/301‑1`  
                                                                                                                                                                   `aAAGAGTCACACTCAGAGTCAACAGACTGGTCCAACGACACTGACTTCTGACAGGATTCTTGATGACTTTCCAGACGACTACGAGAAGTTTCGCTGGAGAGTCCCATTCTAATACGACTCACTAAAGGAGACA‑CACCATGATCGTTTCTGACATCGAAGCTAACGCCCTCTTAGAGAGCGTCACTAAGTTCCACTGCGGGGTTATCTACGACTACTCCACCGCTGAGTACGTAAGCTACCGTCCGAGTGACTTCGGTGCGTATCTGGATGCGCTGGAAGCCGAGGTTGCACGAGGCGGTCT                                                                                                                                           <  1:302217/300‑1`  
                                                                                                                                                                   `aAAGAGTAACACTCAGAGTCAACAGACTGGTCCAACGACACTGACTTCTGACAGGATGCTCGATGACTTTCCAGACGACTACGAGAAGTTGCGCTGGAGGGTCCCATTCTAATACGACGCACTAAAGGAGACA‑CACCATGATCGTTTCTGACATCGAAGCTAACGCCCTCTTAGAGAGCGTCACTAAGTTCCACTGCGGGGTTATCTACGACTACTCCACCGCTGAGTACGTAAGCTACCGTCCGAGTGACTTCGGTGCGTATCTGGATGCGCTGGAAGCCGAGGTTGCACGAGGCAGTCT                                                                                                                                           <  1:218504/300‑1`  
                                                                                                                                                                      `GAGTCACACTCAGAGTCAACAGACTGGTCCAACGACACTGACTTCTGACAGGATTCTTGATGACTTTCCAGACGACTACGAGAAGTTTCGCTGGAGAGTCCCATTCTAATACGACTCACTAAAGGAGACA‑CACCATGATCGTTTCTGACATCGAAGCTAACGCCCTCTTAGAGAGCGTCACTAAGTTCCACTGCGGGGTTATCTACGACTACTCCACCGCTGAGTACGTAAGCTACCGTCCGAGTGACTTCGGTGCGTATCTGGATGCGCTGGAAGCCGAGGTTGCACGAGGCGGTCTTAT                                                                                                                                        >  1:340598/1‑301`  
                                                                                                                                                                        `ggCACACTCAGAGTCAACAGACGGGTCCAACGACACTGACGTCTGACAGGATTCTTGATGACTCTCCAGACGACTACGAGAAGTTTCGCTGGAGAGTCCCATTCTAATACGACTCACTAAAGTAGACA‑CACCATGATCGTTTCGGACATCGAAGACAACGCCCTCTTAGAGAGCGTCACTAAGTTCCACTGCGGGGTTATCTACGACTACTCCACCGCTGAGTACGTAAGCTACCGTCCGAGTGACTTCGGTGCGTATCTGGATGCGCTGGAAGACGAGGTTGCACGAGGCGGTCTTATTG                                                                                                                                      <  1:253593/299‑1`  
                                                                                                                                                                        `aTCACACTCAGAGTCAACAGACTGGTCCAACGACACTGACTTCTGACAGGATTCTTGATGACTTTCCAGACGACTGGCAGAAGTTTCGCTGGAGAGTCCCATTCTAATACGACTCACTAAAGGAGACA‑CACCATGATCGTTTCTGACCTCGAAGCTAACGCCCTCTTAGAGAGCGTCACTAAGTTCCACTGCGGGGTTATCTACGACTACTCCACCGCTGAGTACGTAAGCTACCGTCCGAGTGACTTCGGTGCGTATCTGGATGCGCTGGAAGCCGAGGTTGCACGAGGCGGTCTTATTG                                                                                                                                      <  1:311278/300‑1`  
                                                                                                                                                                        `aTCACACTCAGAGTCAACAGACTGGTCCAACGACACTGACTTCTGACAGGATTCTTGATGACTTTCCAGACGACTACGAGAAGTTTCGCTGGAGAGTCCCATTCTAATACGACTCACTAAAGGAGACA‑CACCATGATCGTTTCTGACATCGAAGCTAACGCCCTCTTAGAGAGCGTCACTAAGTTCCACTGCGGGGTTATCTACGACTACTCCACCGCTGAGTACGTAAGCTACCGTCCGAGTGACTTCGGTGCGTATCTGGATGCGCTGGAAGCCGAGGTTGCACGAGGCGGTCTTATTG                                                                                                                                      <  1:167714/300‑1`  
                                                                                                                                                                        `GTCACACTCAGAGTCAACAGACTGGTCCAACGACACTGACTTCTGACAGGATTCTTGATGACTTTCCAGACGACTACGAGACGTTTCGCTGGAGAGTCCCATTCTAATACGACTCACTAAAGGAGACA‑CACCATGATCGTTTCTGACATCGAAGCTAACGCCCTCTTAGAGAGCGTCACTAAGTTCCACTGCGGGGTTATCTACGACTACTCCACCGCTGAGTAAGTAAGCTACCGTCCGAGTGACTTCGGTGCGTATCTGGATGCGCTGGAAGCCGAGGTTGCACGAGGCGGTCTTATTt                                                                                                                                      >  1:19167/1‑300`  
                                                                                                                                                                        `GTCACACTCAGAGTCAACAGACTGGTCCAAAGACACTGACTTCTGACAGGATTCTTGATGACTTTCCAGACGACTACGAGAAGTTTCGCTGGAGAGTCCCATTCTAATACGACTCACTAAAGGAGACA‑CACCATGATCGTTTCTGACATCGAAGCTAACGCCCTCTTAGAGAGCGTCACTAAGTTCCACTGCGGGGTTATCTACGACTACTCCACCGCTGAGTACGTAAGCTACCGTCCGAGTGACTTCGGTGCGTATCTGGATGCGCTGGAAGCCGAGGTTGTACGAGGCGGTCTTATTG                                                                                                                                      <  1:85984/301‑1`  
                                                                                                                                                                                `CAGAGTCAACAGACTGGTCCAACGACACTGACTTCTGACAGGATTCTTGATGACTTTCCAGACGACTACGAGAAGTTTCGCTGAAGAGTCCCATTCTAATACGACTCACTAAAGGAGACA‑CACCATGATCGTTTCTGACATCGAAGCTAACGCCCTCTTAGAGAGCGTCACTAAGTTCAACTGCGGGGTTATCTACGACTACTCCACCGCTGAGTACGTAAGCTACCGTCCGAGTGACTTCGGTGCGTATCTGGATGCGCTGGAAGCCGAGGTTGCACGAGGCGGTCTTATTGTGTTCCAC                                                                                                                              >  1:135155/1‑301`  
                                                                                                                                                                                   `AGTCAACAGACTGGTCCAACGACACTGACTTCTGACAGGATTCTTGATGACTTTCCAGACGACTACGAGAAGTTTCGCTGGAGAGTCCCATTCTAATACGACTCACTAAAGGAGACA‑CACCATGATCGTTTCTGACATCGAAGCTAACGACCTCTTAGAGAGCGTCACTAAGTTCCACTGCGGGGTTATCTACGACTACTCCACCGCTGAGTACGTAAGCTACCGTCCGAGTGACTTCGGTGCGTATCTGGATGCGCTGGAAGCCGAGGTTGCACGAGGCGGTCTTATTGTGTTCCACAAC                                                                                                                           <  1:233907/301‑1`  
                                                                                                                                                                                           `GACTGGTCCAACGACACTGACTTCTGACAGGATTCTTGATGACTTTCCAGACGACTACGAGAAGTTTCGCTGGAGAGTCCCATTCTAATACGACTCACTAAAGGAGACA‑CACCATGATCGTTTCTGACATCGAAGCTAACGCCCTCTTAGAGAGCGTCACTAAGTTCCACTGCGGGGTTATCTACGACTACTCCACCGCTGAGTACGTAAGCTACCGTCCGAGTGACTTCGGTGCGTATCTGGATGCGCTGGAAGCCGAGGTTGCACGAGGCGGTCTTATTTTGTTCCACAACGGTCACAA                                                                                                                   >  1:71416/1‑301`  
                                                                                                                                                                                            `gaTGGTACTACGACACTGCATGTTGACAGGTTTCTTGATGACTTTACAGACGACTACGAGAAGTTGCGCTGGAGAGTCCCATTCTAATACGACTCACTAAAGGAGACA‑CCCCATGATCGTGTCTGACATCGAAGCTAACGCCCTCTTAGAGAGAGTCACTAAGTTCCACTGCGGGTTTATCTACGACTACTCCTCCGCTGAGTACGTAAGCTACCGTCCGGGTGACGTCGGTGCGTATCTGGATGCGCTGGAAGCCGAGGTTGCACGAGGCGGTCTTATTGTGTTCAACAACGGTCACAAG                                                                                                                  <  1:331743/299‑1`  
                                                                                                                                                                                               `GGTCCAACGACACTGACTTCTGACAGGATTCTTGATGACTTTCCAGACGACTACGAGAAGTTTCGCTGGAGAGTCCCATTCTAATACGACTCACTAAAGGAGACA‑CACCATGATCGTTTCTGACATCGAAGCTAACGCCCTCTTAGAGAGCGTCACTAAGTTCCACTGCGGGGTTATCTACGACTACTCAACCGCTGAGTACGTAAGCTACCATCCGAGTGACTTCGGTGCGTACCTGGATGCGCTGGAAGCCGAGGTTGCACGAGGCGGTCTTATTGTGCTCCACAACGGTCACAAGTAT                                                                                                               >  1:338231/1‑301`  
                                                                                                                                                                                                  `agacaGACACTGACTTCTGACAGGATTCTTGATGACTTTCCAGACGACTACGAGAAGTTTCGCTGGAGAGTCCCATTCTAATACGACTCACTAAAGGAGACA‑CACCATGATCGTTTCTGACATCGAAGCTAACGCCCTCTTAGAGAGCGTCACTAAGTTCCACTGCGGGGTTATCTACGACTACTCCACCGCTGAGTACGTAAGCTACCGTCCGAGTGACTTCGGTGCGTATCTGGATGCGCTGGAAGCCGAGGTTGCACGAGGCGGTCTTATTGTGTTCCACAACGGTCACAAGTATGAC                                                                                                            <  1:289583/296‑1`  
                                                                                                                                                                                                             `GACTTCTGACAGGATTCTTGATGACTTTCCAGACGACTACGAGAAGTTTCGCTGGAGAGTCCCATTCTAATACGACTCACTAAAGGAGACA‑CACCATGATCGTTTCTGACATCGAAGCCAACGCCCTCTTAGAGAGCGTCACTAAGTTCCACTGCGGGGTTATCTACGACTACTCCACCGCTGAGTACGTAAGCTACCGTCCGAGTGACTTCGGTGCGTATCTGGATGCGCTGGAAGCCGAGGTTGCACGAGGCGGTCTTATTGTGTTCCACAACGGTCACAAGTATGACGTTCCTGCATT                                                                                                 >  1:161721/1‑301`  
                                                                                                                                                                                                               `CTTCTGACAGGATTCTTGATGACTTTCCAGACGACTACGAGAAGTTTCGCTGGAGAGTCCCATTCTAATACGACTCACTAAAGGAGACA‑CACCATGATCGTTTCTGACATCGAAGCTAACGCCCTCTTAGAGAGCGTCACTAAGTTCCACTGCGGGGTTATCTACGACTACTCCACCGCTGAGTACGTAAGCTACCGTCCGAGTGACTTCGGTGCGTATCTGGATGCGCTGGAAGCCGAGGTTGCACGAGGCGGTCTTATTGTGTTCCACAACGGTCACAAGTATGACGTTCCTGCATTGA                                                                                               >  1:315954/1‑301`  
                                                                                                                                                                                                                             `CTTGATGACTTTCCAGACGACTACGAGAAGTTTCGCTGGAGAGTCCCATTCTAATACGACTCACTAAAGGAGACA‑CACCATGATCGTTTCTGACATCGAAGCTAACGCCCTCTTAGAGAGCGTCACTAAGTTCCACTGCGGGGTTATCTACGACTACTCCACCGCTGAGTACGTAAGCTACCGTCCGAGTGACTTCGGTGCGTATCTGGATGCGCTGGAAGCCGAGGTTGCACGAGGCGGTCTTATTGTGTTCCACAACGGTCACAAGTATGACGTTCCTGCATctgtctcttatacacat                                                                                 >  1:13271/1‑284`  
                                                                                                                                                                                                                                     `CTTTCCAGACGACTACGAGAAGTTTCGCTGGAGAGTCCCATTCTAATACGACTCACTAAAGGAGACA‑CACCATGATCGTTTCTGACATCGAAGCTAACGCCCTCTTAGAGAGCGTCACTAAGTTCAACTGCGGGGTTATCTACGACTACTCCACCGCTGAGTACGTAAGCTACCGTCCGAGTGACTTCGGTGCGTATCTGGATGCGCTGGAAGCCGAGGTTGCACGAGGCGGTCTTATTGTGTTCCACAACGGTCACAAGTATGACGTTCCTGCATTGACCAAACTGGCAAAGTTGCAATT                                                                         >  1:313766/1‑301`  
                                                                                                                                                                                                                                               `GACTACGAGAAGTTTCGCTGGAGAGTCCCATTCTAAAACGACTCACTAAAGGAGACA‑CACCATGATCGTTTCTGACATCGAAGCTAACGCCCTCTTAGAGAGCGTCACTAAGTTCCACTGCGGGGTTATCTACGACTACTCCACCGCTGAGTACGTAAGCTACCGTCCGAGTGACTTCGGTGCGTATCTGGATGCGCTGGAAGCCGAGGTTGCACGAGGCGGTCTTATTGTGTTCCACAACGGTCACAAGTATGACGTTCCTGCATTGACCAAACTGGCAAAGTTGCAATTGAACCGAGAG                                                               <  1:166938/301‑1`  
                                                                                                                                                                                                                                                         `agacagGCTGGAGAGTCCCATTCTAATACGACTCACTAAAGGAGACA‑CACCATGATCGTTTCTGACATCTAAGCTAACGCCCTCTTAGAGAGCGTCACTGAGTTCCACTGCGGGGTTATCTACGACTACTCCACCGCTGAGTACGTAAGCTACCGTCCGAGTGACTTCGGTGCGTATCTGGATGCGCTGGAAGCCGAGGTTGCACGAGGCGGTCTTATTGTGTTCCACAACGGTCACAAGTATGACGTTCCTGCATTGACCAAACTGGCAAAGTTGCAATTTAACCGAGAGTTCCACCTTC                                                     <  1:100648/295‑1`  
                                                                                                                                                                                                                                                            `TTCGCTTGAGAGTCCCATTCTAATACGACTCACTAAAGGAGACA‑CACCATGATCGTTTCTGACATCGAAGCTAACGCCCTCTTAGAGAGCGTCACTAAGTTCCACTGCGGGGTTATCTACGACTACTCCACCGCTGAGTACGTAAGCTACCGTCCGAGTGACTTCGGTGCGTATCTTGATGCGCTGGAAGCCGAGGTTGCACGAGGCGGTCTTATTTTGTTCCACAACGGTCACAAGTATGACGTTCCTGCATTGACCAAACTGGCAAAGTTGCAATTGAACCGAGAGTTCCACCTTCCTC                                                  >  1:251944/1‑301`  
                                                                                                                                                                                                                                                                    `acAGTCCCAGTCTAAGACGACTCATTAAAGGAGACA‑AACCAGGATCGTTGTTGACATCGAAGCTAACGCCATCTTAGAGAGCGTAACTAAGTTCCACTGCGGGGTTATCTACGACTACTCCACCGCTGAGTACGTAAGCTACCGTCCGAGTGACTTCGGTGCGTATCTGGATGCACTGGAAGCCGAGGTTGCACGAGGCGGTCTTATTGTGTTCCACAACGGTCACAAGTATGACGTTCCTGCATTGACCAAACTGGCAAAGTTGCAATTGAACCGAGAGTTCCACCTTCCTCGTGAGAAC                                          <  1:75720/299‑1`  
                                                                                                                                                                                                                                                                       `GTCCCATTCTAATACGACTCACTAAAGGAGACA‑CACCATGATCGTTTCTGACATCGAAGCTAACGCCCTCTTAGAGAGCGTCACTAAGTTCCACTGCGGGGTTATCTACGACTACTCCACCGCTGAGTACGTAAGCTACCGTCCGAGTGACTTCGGTGCGTATCTGGATGCGCTGGAAGCCGAGGTTGCACGAGGCGGTCTTATTGTGTTCCACAACGGTCACAAGTATGACGTTCCTGCATTGACCAAACTGGCAAAGTTGCACTTGAACCGAGAGTTCCACCTTCCTCGTGAGAACTGT                                       >  1:333567/1‑301`  
                                                                                                                                                                                                                                                                       `GTCCCATTCTAATACGACTCACTAAAGGAGACA‑CACCATGATCGTTTCTGACATCGAAGCTAACGCCCTCTTAGAGAGCGTCACTAAGTTCCACTGCGGGGTTATCTACGACTACTCCACCGCTGAGTACGTAAGCTACCGTCCGAGTGACTTCGGTGCGTATCTGGATGCGCTGGAAGCCGAGGTTGCACGAGGAGGTCTTATTGTGTTACACAACGGTCACAAGTATGACGTTCCTGCATTGACCAAACTGGCAAAGTTGCAATTGAACCGAGAGTTCCACCTTCCTCGTGAGAACTGT                                       >  1:125987/1‑301`  
                                                                                                                                                                                                                                                                          `CCATTCTAATACGACTCACTAAAGGAGACA‑CACCATGATCGTTTCTGACATCGAAGCTAACGCCCTCTTAGAGAGCGTCACTAAGTTCCACTGCGGGGTTATCTACGACTACTCCACCGCTGAGTACGTAAGCTACCGTCCGAGTGACTTCGGTGCGTATCTGGATGCGCTGGAAGCCGAGGTTGCACGAGGCGGTCTTATTGTGTTCCACAACGGTCACAAGTATGACGTTCCTGCATTGACCAAACTGGCAAAGTTGCAATTGAACCGAGAGTTCCACCTTCCTCGTGAGAACTGTATT                                    >  1:28196/1‑301`  
                                                                                                                                                                                                                                                                            `ATTCTAATACGACTCACTAAAGGAGACA‑CACCATGATCGTTTCTGACATCGAAGCTAACGCCCTCTTATAGAGCGTCACTAAGTTCCACTGCGGGGTTATCTACGACTACTCCACCGCTGAGTACGTAAGCTACCGTCCGAGTGACTTCGGTGCGTATCTGGATGCGCTGGAAGCCGAGGTTGCACGAGGCGGTCTTATTGTGTTCCACAACGGTCACAAGTATGACGTTCCTGCATTGTCCAAACTGGCAAAGTTGCAATTGAACCGAGAGTTCCACCTTCCTCGTGAGAACTGAATTGA                                  >  1:207940/1‑301`  
                                                                                                                                                                                                                                                                             `TTCTAATACGACTCACTAAAGGAGACA‑CACCATGATCGTTTCTGACATCGAAGCTAACGCCCTCTTAGAGATCGTCACTAAGTTCCACTGCGGGGTTATCTACGACTACTCCACCGCTGAGTACGTAAGCTACCGTCCGAGTGACTTCGGTGCGTATCTGGATGCGCTGTAAGCCGAGGTTGCACGAGGCGGTCTTATTTTGTTCCACAACGGTCACAAGTATGACGTTCCTGCATTGACCAAACTGGCAAAGTTGCAATTGAACCGAGAGTTCCACCTTCCTCGTGAGAACTGTATTGAC                                 >  1:265956/1‑301`  
                                                                                                                                                                                                                                                                             `TTCTAATACGACTCACTAAAGGAGACA‑CACCATGATCGTTTCTGACATCGAAGCTAACGCCCTCTTAGAGAGCGTCACTAAGTTCCACTGCGGGGTTATCTACGACTACTCCACCGCTGAGTACGTAAGCTACCGTCCGAGTGACTTCGGTGCGTATCTGGATTCGCCGGAAGCCGAGGTTGCACGAGGCGGTCTTATTGTGTTCCACAACGGTCACAAGTATTACGTTCCTGCATTGACCAAACTGGCAAAGTTGCAATTGAACCGAAAGTTCCACCTTCCTCCTTAGAACTGTATTTAC                                 >  1:336635/1‑301`  
                                                                                                                                                                                                                                                                             `TTCTAATACGACTCACTAAAGGAGACA‑CACCATGATCGTTTCTGACATCGAAGCTAACGCCCTCTTAGAGAGCGTCACTAAGTTCCACTGCGGGGTTATCTACGACTACTCCACCGCTGAGTACGTAAGCTACCGTCCGAGTGACTTCGGTGCGTATCTGGATGCGCTGGAAGCCGAGGTTGCACGAGGCGGTCTTATTGTGTTCCACAACGGTCACAAGTATGACGTTCCTGCATTGACCAAACTGGCAAAGTTGCAATTGAACCGAGAGTTCCACCTTCCTCGTGAGAACTGTATTGAa                                 >  1:18646/1‑300`  
                                                                                                                                                                                                                                                                             `TTCTAATACGACTCACTAAAGGAGACA‑CACCATGATCGTTTCTGACATCGAAGCTAACGCCCTCTTAGAGAGCGTCACTAAGTTCCACTGCGGGGTTATCTACGACTACTCCACCGCTGAGTACGTAAGCTACCGTCCGAGTGACTTCGGTGCGTATCTGGATGCGCTGGAAGCCGAGGTTGCACGAGGCGGTCTTATTGTGTTCCACAACGGTCACAAGTATGACGTTCCTGCATTGACCAAACTGGCAAAGTTGCAATTGAACCGAGAGTTCCACCTTCCTCGTGAGAACTGTATTGAC                                 >  1:96991/1‑301`  
                                                                                                                                                                                                                                                                             `TTCTAATACGACTCACTAAAGGAGACA‑CACCATGATCGTTTCTGACATCGAAGCTAACGCCCTCTTAGAGAGCGGCACTAAGTTCCACTGCTGGGTTATCTACGACTACGCCACCGCTGAGTACGTAAGCTACCGTCCGAGTGACTTCGGTGCGTATCTGGATGCGCTGGAAGCCGAGGTTGCACGAGGCGGTCTTATTGTGTTCCACAACGGTCACAAGTATGACGTTCCTGCATTGACCAAACTGGCAAAGTTGCAATTGAACCGAGAGTTCCACCTTCCTCGTGAGAACTGTATTGAC                                 <  1:286334/301‑1`  
                                                                                                                                                                                                                                                                               `CTAATACGACTCACTAAAGGAGACA‑CACCATGATCGTTTCTGACATCGAAGCTAACGCCCTCTTAGAGAGCGTCACTAAGTTCCACTGCGGGGTTATCTACGACTACTCCACCGCTGAGTACGTAAGCTACCGTCCGAGTGACTTCGGTGCGTATCTGGATGCGCTGGAAGCCGAGGTTGCACGAGGCGGTCTTATTGTGTTCCACAACGGTCACAAGTATGACGTTCCTGCATTGACCAAACTGGCAAAGTTGCAATTGAACCGAGAGTTCCACCTTCCTCGTGAGAACTGTATTGACAC                               <  1:210414/301‑1`  
                                                                                                                                                                                                                                                                                 `ttttatgaatcaaacaagataaaccattATGATCGTTTCCGACATCGAACCTCACGCCCTTCTAGAGTGCCTTACTAAGTTCCACTGCGGGGTTATCTACGACTACGCCACCGCTGAGTACGTAAGCTACCGTCCGAGTGACTTCGGTGCGTATCTGGATGCGCTGGAAGCCGAGGTTGCACGAGGCGGTCTTATTGTGTTCCACAACGGTCACAAGTATGACGTTCCTGCATTGACCAAACTGGCAAAGTTGCAATTGAACCGTGAGTTCCACCTTCCTCGTGAGAACTGTATTGACACC                              <  1:29474/273‑1`  
                                                                                                                                                                                                                                                                                    `ACGACTAACTAAAGGAGACA‑CACCATGATCGTTTCTGACATCGAAGCTAACGCCATCTTAGAGAGCGTCACTAAGTTCCACTGCGGGGTTATCTACGACTACTCCACCGCTGAGTACGTAAGCTACCGTCCGAGTGACTTCGGTGCGTATCTGGATGCGCTGGAAGCCGAGGTTGCACGAGGCGGTCTTATTGTGTTCCACAACGGTCACAAGTATGACGTTCCTGCATTGACCAAACTGGCAAAGTTGCAATTGAACCGAGAGTTCCACCTTCCTCGTGAGAACTGTATTGACACACTTG                          <  1:66996/301‑1`  
                                                                                                                                                                                                                                                                                     `gagatgtgtatcaGAGACAGCACCATGATCGTTTCTGACATCGAAGCTAACGCCCTCTTAGAGAGCGTCACTAAGTTCCACTGCGGGGTTATCTACGACTACTCCACCGCTGAGTACGTAAGCTACCGTCCGAGTGACTTCGGTGCGTATCTGGATGCGCTGGAAGCCGAGGTTGCACGAGGCGGTCTTATTGTGTTCCACAACGGTCACAAGTATGACGTTCCTGCATTGACCAAACTGGCAAAGTTGCAATTAAACCGAGAGTTCCACCTTCCTCGTGAGAACTGTATTGACACCCTTG                          <  1:244728/288‑1`  
                                                                                                                                                                                                                                                                                       `cagCACTAAAGGAGACA‑CACCATGATCGTTTCTGACATCGAAGCTAACGCCCTCTTAGAGAGCGTCACTAAGTTCCACTGCGGGGTTATCTACGACTACTCCACCGCTGAGTACGTAAGCTACCGTCCGAGTGACTTCGGTGCGTATCTGGATGCGCTGGAAGCCGAGGTTGCACGAGGCGGTCTTATTGTGTTCCACAACGGTCACAAGTATGACGTTCCTGCATTGACCAAACTGGCAAAGTTGCAATTGAACCGAGAGTTCCACCTTCCTCGTGAGAACTGTATTGACACCCTTGTGT                       <  1:3516/298‑1`  
                                                                                                                                                                                                                                                                                          `CACTAAAGGAGACA‑CACCATGATCGTTTCTGACATCGAAGCTAACGCCCTCTTAGAGAGCGTCACTAAGTTCCACTGCGGGGTTATCTACGACTACTCCACCGCTGAGTACGTAAGCTACCGTCCGAGTGACTTCGGTGCGTATCTGGATGCGCTGGAAGCCGAGGTTGCACGAGGCGGTCTTATTGTGTTCCACAACGGTCACAAGTATGACGTTCCTGCATTGACCAAACTGGCAAAGTTGCAATTGAACCGAGAGTTCCATCTTCCTCGTGAGAACTGTATTGACACCCTTGTGTTGT                    >  1:250870/1‑301`  
                                                                                                                                                                                                                                                                                          `CACTAAAGGAGACA‑CACCATGATCGTTTCTGACATCGAAGCTAACGCCCTCTTAGAGAGCGTCACTAAGTTCCACTGCGGGGTTATCTACGACTACTCCACCGCTGAGTACGTAAGCTACCGTCCGAGTGACTTCGGTGCGTATCTGGATGCGCTGGAAGCCGAGGTTGCACGAGGCGGTCTTATTGTGTTCCACAACGGTCACAAGTATGACGTTCCTGCATTGACCAAACTGGCAAAGTTGCAATTGAACCGAGAGTTCCACCTTCCTCGTGAGAACTGTATTGACACCCTTGTGTTGT                    >  1:273941/1‑301`  
                                                                                                                                                                                                                                                                                            `CTATAGGAGACA‑CACCATGATCGTTTCTGACATCGAAGCTAACGCCCTCTTAGAGAGCGTCACTAAGTTCCACTGCGGGGTTATCTACGACTAATCCACCGCTGAGTACGTAAGCTACCGTCCGAGTGACTTCGGTGCGTATCTGGATGCGCTGGAAGCCGAGGTTGCACGAGGCGGCCTTATTGTGTTCCACAACGGTCACAAGTATGACGTTCCTGCATTGACAAAACTGGCAACGTTGCAATTACACCGAGAGTTCCACCTTCCTCGTGAGAACTGTATTGACACCCTTGTGTTGTCc                  >  1:198431/1‑300`  
                                                                                                                                                                                                                                                                                            `CTAAAGGAGACA‑CCCCATGATCGTTCCTGACATCGAAGCTAACGCCCTCTTAGAGAGCGTCACTAAGTTCCACTGCGGGGTTATCTACGACTACTCCACCGCTGAGTACGTAAGCTACCGTCCGAGTGACTTCGGTGCGTATCTGGATGCGCTGGAAGCCGATGTTGCACGAGGCGGTCTTATTGTGTTCCACAACGGTCACAAGTATGACGTTCCTGCATTGACCAAAATGGCAAAGTTGTAATTGAACCGAGAGTTCCACCTTCCTCGTGAGAACTGTATTGACACCCTTGTGTTGTCA                  <  1:179978/301‑1`  
                                                                                                                                                                                                                                                                                               `AAGGAGACA‑CACCATGATCGTTTCTGACATCGAAGCTAACGCCCTCTTAGAGAGCGTCACTAAGTTCCATTGCGGGGTTATCTACGACTACTCCACCGCTGAGTACGTAAGCTACCGTCCGAGTGACTTCGGTGCGTATCTGGATGCGCTGGAAGCCGAGGTTGCACGAGGCGGTCTTATTGTGTTCCACAACGGTCACAAGTATGACGTTCCTGCATTGACCAAACTGGCAAAGTTGCAATTGAACCGAGAGTTCCACCTTCCTCGTGAGAACTGTATTGACACCCTTGTGTTGTCACGT               >  1:46994/1‑301`  
                                                                                                                                                                                                                                                                                                 `GGAGACA‑CACCATGATCGTTTCTGACATCGAAGCTAACGCCCTCTTAGAGAGCGTCACTAAGTTCCACTGCGGGGTTATCTACGACTACTCCACCGCTGAGTACGTAAGCTACCGTCCGAGTGACTTCGGTGCGTATCTGGATGCGCTGGAAGCCGAGGTTGCACGAGGCGGTCTTATTGTGTTCCACAACGGTCACAAGTATGACGTTCCTGCATTGACCAAACTGGCAAAGTTGCAATTGAACCGAGAGTTCCACCTTCCTCGTGAGAACTGTATTGACACCCTTGTGTTGctgtctct             >  1:12116/1‑293`  
                                                                                                                                                                                                                                                                                                  `GAGACA‑CACCATGATCGTTTCTGACATCGAAGCTAACGCCCTCTTAGAGAGCGTCACTAAGTTCCACTGCGGGGTTATCTACGACTACTCCACCGCTGAGTACGTAAACTACGGTCCGAGTGACTTCGGTGCGTATCTGGATGCGCTGGAAGCCGAGGTTGCACGAGGCGGTCTTATTGTGTTCCACAACGGTCACAAGTATGACGTTCATGCATTGACCAAACTGGCAAAGTTGCAATTGAACCGAGAGTTCCACCTTCCTAGTGAGAACTGTATTAACACCCTTGTGTTGTCACGTTTG            >  1:113091/1‑301`  
                                                                                                                                                                                                                                                                                                      `CA‑CACCATGATCGTTTCTGACATCGAAGCTAACGCCCTCTTAGAGTGCGTCACTAAGTTCCACTGCGGGGTTATCTACGACTACTCCACCGCTGAGTACGTAAGCTACCGTCCGAGTGACTTCGGTGCGTATCTGGATGCGCTGGAAGCCAAGGTTGCACGAGGCGGTCTTATTGTGTTCCACAACGGTCACAAGTATGACGTTCCTGCATTGACCAAACTGGCAAAGTTGCAATTGAACCGAGAGTTCCACCTTCCTCGTGAGAACTGTATTGACACCCTTGTGTTGTCACGTTTGATTC        <  1:226344/301‑1`  
                                                                                                                                                                                                                                                                                                      `CA‑CACCATGATCGTTTCTGACATCGAAGCTAACGCCCTCTTAGAGAGCGTCACTAAGTTCCACTGCGGGGTTATCTACGACTACTCCACCGCTGAGTACGTAAGCTACCGTCCGAGTGACTTCGGTGCGTATCTGGATGCGCTGGAAGCCGAGGTTGCACGAGGCGGTCTTATTGTGTTCCACAACGGTCACAAGTATGACGTTCCTGCATTGACCAAACTGGCAAAGTTGCAATTGAACAGAGAGTTCCACCTTCCTCGTGAGAACTGTATTGACACCCTTGTGTTGTCACGTTTGATTC        <  1:148884/301‑1`  
                                                                                                                                                                                                                                                                                                         `CACCATGATCGTTTCTGACATCGAAGCTAACGCCCTCTTAGATAGCGTCACTAAGTTCCACTGCGGGGTTATCTACGACTACTCCACCGCTGAGTACGTAAGCTACCGTCCGAGTGACTTCGGAGCGTATCTGGATGCGCTGGAAGCCGAGGTTGCACGAGGCGGTCTTATTGTGTTCCACAACGGTCACAAGTATGACGTTCCTGCATTGACCAAACTGTCAAAGTTGCAATTGAACCGAGAGTTCCACCTTCCTCGTGAGAACTGTTTTGACACCCTTGTGTTGTCACGCTTTATTCAT      >  1:59991/1‑301`  
                                                                                                                                                                                                                                                                                                         `CACCATGATCGTTTCTGACATCGAAGCTAACGCCCTCTTAGAGAGCGTCACTAAGTTCCACTGCGGGGTTATCTACGACTACTCCACCGCTGAGTACGTAAGCTACCGTCCGAGTGACTTCGGTGCGTATCTGGATGCGCTGGAAGCCGAGGTTGCACGAGGCGGTCTTATTGTGTTCCACAACGGTCACAAGTATGACGTTCCTGCATTGACCAAACTGGCAAAGTTGCAATTTAACCTAGAGTTCCACCTTCCTCGTGAGAACTGTATTGACACCCTTGTGTTGTCACGTTTGATTCAT      >  1:158290/1‑301`  
                                                                                                                                                                                                                                                                                                           `CCATGATCGTTTCTGACATCGAAGCTAACGCCCTCTTAGAGAGCGTCACTAAGTTCCACTGCGGGGTTATCTACGACTACTCCACCGCTGAGTACGTAAGCTACCGTCCGAGTGACTTCGGTGCGTATCTGGATGCGCTGGAAGCCGAGGTTGCACGAGGCGGTCTTATTGTGTTACACAACGGTCACAAGTATGACGTTCCTGCATTGACCAAACTGGCAAAGTTGCAATTGAACCGAGAGTTCCACCTTCCTCATGAGAACTGTATTGACACtgtctcttatacacatctccgagccca    >  1:323234/1‑274`  
                                                                                                                                                                                                                                                                                                            `aATGATCGTTTCTGACATCGAAGCTAACGCCCTCTTAGAGAGCGTCACTAAGTTCCACTGCGGGGTTATCTACGACTACTCCACCGCTGAGTACGTAAGCTACCGTCCGAGTGACTTCGGTGCGTATCTGGATGCGCTGGAAGCCGAGGTTGCACGAGGCGGTCTTATTGTGTTCCACAACGGTCACAAGTATGACGTTCCTGCATTGACCAAACTGGCAAAGTTGCAATTGAACCGAGAGTTCCACCTTCCTCGTGAGAACTGTATTGACACCCTTGTGTTGTCACGTTTGATTCATTCC   <  1:9852/300‑1`  
                                                                                                                                                                                                                                                                                                            `aATGATCGTTTCTGACATCGAAGCCAACGCCCTCTTAGATAGCGTCACTAAGTTCCACTGCGGGGTGATCTACGACTACTCCACCGCTGAGTACGTAAGCTACCGTCCGAGTGACTTCGGTGCGTATCTGGATGCGCTGGAAGCCGAGGTTGCACGAGGCGGTCTTATTGTGTTCCACAACGGTAACAAGTATGACGTTCCTGCATTGACCAAACTGGCAAAGTTGCAATTGAACCGAGAGTTCCACCTTCCTCGTGAGAACTGTATTGACACCCTTGTGTTGTCACGTTTGATTCATTCC   <  1:147401/300‑1`  
                                                                                                                                                                                                                                                                                                            `CATGATCGTTTCTGACATCGAAGCTAACGCCCTCTTAGAGAGCGTCACTAAGTTCCACTGCGGGGTTATCTACGACTACTCCACCGCTGAGTACGTAAGCTACCGTCCGAGTGACTTCGGTGCGTATCTGGATGCGCTGGAAGCCGAGGTTGCACGAGGCGGTCTTATTGTGTTCCACAACGGTCACAAGTATGACGTTCCTGCATTGACCAAACTGGCAAAGTTGCAATTGAACCGAGAGTACCACCTTCCTCGTGAGAACTGTATTGACACCCTTGTGTTGTCACGTTTGATTCATTCC   <  1:88983/301‑1`  
                                                                                                                                                                                                                                                                                                            `CATGATCATTTCTGACATCGAAGCTAACGCCCTCTTAGAGAGCGTCACTAAGTTCCACTGCTGGGTTATCTACGACTACTCCACCGCTGAGTACGTAAGCTACCGTCCGAGTGACTTCGGTGCGTATCTGGATGCGCTGGAAGCCGAGGTTGCACGAGGCGGTCTTATTGTGTTCCACAACGGTCACAAGTATGACGTTCCTGCATTGACCAAACTGGCAAAGTTGCAATTTAACCGAGAGTTCCACCTTCCTCGTGAGAACTGTATTGACACCCTTGTGTTGTCACGTTTGATTCATTCC   <  1:215615/301‑1`  

                                                                                                                                                                                                                                                                                                                                                                                                                                                                                                                                                                                                                            
`TGATACTATTATTGCCCTTGAGCGTAATCAGCAAGGCGATATGCCTAACCTTGTCCTCGTTCGTATTCTCAAGTGCCGCTTTACTGGTGATACTGGTATCGCTGGCTACATGGAATACAACAAGGAAACCGGATGGCTTGAACCATCAAGTTACTCAGGGGAAGAAGAGTCACACTCAGAGTCAACAGACTGGTCCAACGACACTGACTTCTGACAGGATTCTTGATGACTTTCCAGACGACTACGAGAAGTTTCGCTGGAGAGTCCCATTCTAATACGACTCACTAAAGGAGACA‑CACCATG‑‑‑‑‑‑‑‑‑‑‑‑‑‑‑‑‑‑‑‑‑‑‑‑‑‑‑‑‑‑‑‑‑‑‑‑‑‑‑‑‑‑‑‑‑‑‑‑‑‑‑‑‑‑‑‑‑‑‑‑‑‑‑‑‑‑‑‑‑‑‑‑‑‑‑‑‑‑‑‑‑‑‑‑‑‑‑‑‑‑‑‑‑‑‑‑‑‑‑‑‑‑‑‑‑‑‑‑‑‑‑‑‑‑‑‑‑‑‑‑‑‑‑‑‑‑‑‑‑‑‑‑‑‑‑‑‑‑‑‑‑‑‑‑‑‑‑‑‑‑‑‑‑‑‑‑‑‑‑‑‑‑‑‑‑‑‑‑‑‑‑‑‑‑‑‑‑‑‑‑‑‑‑‑‑‑‑‑‑‑‑‑‑‑‑‑‑‑‑‑‑‑‑‑‑‑‑‑‑‑‑‑‑‑‑‑‑‑‑‑‑‑‑‑‑‑‑‑‑‑‑‑‑‑‑‑‑‑‑‑‑‑‑‑‑‑‑‑‑‑‑‑‑‑‑‑‑‑‑‑‑‑‑‑‑‑‑‑‑‑‑‑‑‑‑‑‑‑‑‑‑‑‑‑‑‑‑‑‑‑‑‑‑‑‑‑‑‑  >  NC_001604.1/13052‑13354`  
`‑‑‑‑‑‑‑‑‑‑‑‑‑‑‑‑‑‑‑‑‑‑‑‑‑‑‑‑‑‑‑‑‑‑‑‑‑‑‑‑‑‑‑‑‑‑‑‑‑‑‑‑‑‑‑‑‑‑‑‑‑‑‑‑‑‑‑‑‑‑‑‑‑‑‑‑‑‑‑‑‑‑‑‑‑‑‑‑‑‑‑‑‑‑‑‑‑‑‑‑‑‑‑‑‑‑‑‑‑‑‑‑‑‑‑‑‑‑‑‑‑‑‑‑‑‑‑‑‑‑‑‑‑‑‑‑‑‑‑‑‑‑‑‑‑‑‑‑‑‑‑‑‑‑‑‑‑‑‑‑‑‑‑‑‑‑‑‑‑‑‑‑‑‑‑‑‑‑‑‑‑‑‑‑‑‑‑‑‑‑‑‑‑‑‑‑‑‑‑‑‑‑‑‑‑‑‑‑‑‑‑‑‑‑‑‑‑‑‑‑‑‑‑‑‑‑‑‑‑‑‑‑‑‑‑‑‑‑‑‑‑‑‑‑‑‑‑‑‑‑‑‑‑‑‑‑‑‑‑‑‑‑‑‑‑‑‑‑‑‑‑‑‑‑‑‑‑‑‑‑‑‑‑‑‑‑‑‑‑‑‑‑‑‑‑‑‑‑‑‑‑atgATCGTTTCTGACATCGAAGCTAACGCCCTCTTAGAGAGCGTCACTAAGTTCCACTGCGGGGTTATCTACGACTACTCCACCGCTGAGTACGTAAGCTACCGTCCGAGTGACTTCGGTGCGTATCTGGATGCGCTGGAAGCCGAGGTTGCACGAGGCGGTCTTATTGTGTTCCACAACGGTCACAAGTATGACGTTCCTGCATTGACCAAACTGGCAAAGTTGCAATTGAACCGAGAGTTCCACCTTCCTCGTGAGAACTGTATTGACACCCTTGTGTTGTCACGTTTGATTCATTCCA  >  NC_001604.1/14356‑14653`

  

Alignment Legend

Aligned base mismatch/match (shaded by quality score): `ATCG/ATCG` `< 3 ≤` `ATCG/ATCG` `< 11 ≤` `ATCG/ATCG` `< 23 ≤` `ATCG/ATCG` `< 35 ≤` `ATCG/ATCG` `< 38 ≤` `ATCG/ATCG`

Unaligned base: `atcg`    Masked matching base: `atcg`    Alignment gap: `‑`    Deleted base: `‑`

Reads not counted as support for junction

read\_name Not counted due to insufficient overlap past the breakpoint.

read\_name Not counted due to not crossing MOB target site duplication.
